# Integrated Network Pharmacology, Single‐Cell Transcriptomics Unveil the Mechanistic Role of Morusin in Aortic Dissection

**DOI:** 10.1111/jcmm.70971

**Published:** 2026-01-02

**Authors:** Zhaomeng Wang, Haoran Zhang, Zhanxiong Xie, Yukun Xiang, Yiwen Fu, Zixun Wang, Haiqing Jiao, Nan Lin, Chenguang Niu, Chao Jiang, Lemin Zheng

**Affiliations:** ^1^ Beijing Tiantan Hospital China National Clinical Research Center for Neurological Diseases, Advanced Innovation Center for Human Brain Protection, Beijing Institute of Brain Disorders, the Capital Medical University Beijing China; ^2^ The Second School of Clinical Medicine Southern Medical University Guangzhou Guangdong China; ^3^ Department of Cardiology, Affiliated Nanping First Hospital Fujian Medical University Nanping Fujian China; ^4^ The Institute of Cardiovascular Sciences and Institute of Systems Biomedicine, School of Basic Medical Sciences, State Key Laboratory of Vascular Homeostasis and Remodeling, NHC Key Laboratory of Cardiovascular Molecular Biology and Regulatory Peptides, Beijing Key Laboratory of Cardiovascular Receptors Research, Health Science Center Peking University Beijing China; ^5^ Beijing Tiantan Hospital, China National Clinical Research Center for Neurological Diseases, Advanced Innovation Center for Human Brain Protection, Beijing Institute of Brain Disorders The Capital Medical University Beijing China; ^6^ School of Stomatology Henan University Kaifeng Henan China; ^7^ Department of Cardiology Fujian Provincial Hospital Fuzhou Fujian China; ^8^ Department of Cardiology, Beijing Anzhen Hospital, Capital Medical University, Engineering Research Center of Medical Devices for Cardiovascular Diseases Ministry of Education, National Clinical Research Center for Cardiovascular Diseases Beijing China

**Keywords:** aortic dissection, IL‐17 signalling pathway, molecular docking, Morusin, network pharmacology, single‐cell transcriptomics

## Abstract

Aortic dissection is a life‐threatening cardiovascular emergency with limited pharmacological options. This study focuses on elucidating the multi‐target and multi‐pathway mechanisms through which morusin mitigates aortic dissection progression, integrating network pharmacology, single‐cell transcriptomics and experimental validation. Multi‐database analysis identified 281 morusin targets and 1741 ad‐related genes, with 84 overlaps. Enrichment analyses highlighted IL‐17, HIF‐1 and MAPK signalling pathways as potential regulatory hubs. Protein–protein interaction network analysis identified seven key targets, all showing high binding affinity to morusin in molecular docking. Single‐cell transcriptomics revealed cell‐type‐specific dysregulation, notably MAPK8 upregulation in fibroblasts and immune cells. In vitro, morusin dose‐dependently inhibited AngII‐induced vascular smooth muscle cell proliferation and modulated IL‐17 pathway gene expression. In vivo, morusin attenuated aortic dilation and reduced morbidity and mortality in a BAPN‐induced AD mouse model. These findings suggest that morusin mitigates AD progression by targeting key inflammatory and apoptotic pathways, supporting its potential as a multi‐target therapeutic candidate.

## 1 Introduction

Aortic dissection (AD) is a life‐threatening cardiovascular emergency caused by intimal tearing or intramural haemorrhage that separates the aortic media, forming a ‘false lumen’ distinct from the ‘true lumen.’ This process may lead to rupture, ischemia or malperfusion, contributing to a mortality rate exceeding 30% within 2 weeks of onset [1, 2]. Despite advances in surgical management, effective pharmacological therapies remain unavailable and AD continues to be a major cause of sudden death. The incidence is approximately 3–5 cases per 100,000 person‐years in the general population, with higher rates among older men, particularly in Asian countries such as China and Japan [3]. Hypertension (present in 80% of patients), connective‐tissue disorders (e.g., Marfan and Loeys‐Dietz syndromes), atherosclerosis, smoking and ageing are key risk factors, while metabolic abnormalities, including elevated plasma succinate, have recently been linked to disease progression [4].

AD is clinically classified by the Stanford and DeBakey systems [5], with the Stanford system predominating for its distinction between Type A lesions requiring emergent surgical repair due to ascending aortic involvement and Type B lesions managed through pharmacological therapy or thoracic endovascular aortic repair when confined to the descending aorta [6]. Pathologically, hypertension remains the dominant driver, increasing wall fragility through hemodynamic stress, angiotensin II–induced activation of vascular smooth muscle cells, and macrophage‐mediated apoptosis, ultimately promoting inflammatory cytokine release (IL‐6, TNF‐α, MCP‐1) and matrix degradation [7, 8].

Morusin, a prenylated flavonoid isolated from the root bark of *Morus* species, exhibits notable antioxidant activity and lipophilic properties. Studies have demonstrated that morusin exerts its therapeutic potential in various diseases by targeting key signalling molecules such as COX‐2, LOXs, EGFR, STAT3 and NF‐κB [9]. Through the regulation of inflammatory responses, apoptosis, metabolic reprogramming and autophagy, morusin plays a crucial role in disease modulation. In recent years, its pharmacological effects have been extensively investigated in the contexts of diabetes [10], inflammatory disorders [11], neurodegenerative diseases [12] and cancer [13]. In diabetic mice, morusin lowers glucose and lipid peroxidation while protecting β‐cells [14], and in tumours it suppresses NF‐κB/STAT3 signalling, induces apoptosis and reduces stemness marker expression (Oct 4, Sox2) [15]. It also alleviates inflammation in colitis and arthritis models by reducing TNF‐α, IL‐1β, NO and PGE2 [16]. In cardiovascular contexts, morusin modulates the Ccnd1/Trim25/Nrf2 axis to suppress valvular interstitial cell senescence and calcification, thereby improving valve function and reducing oxidative stress in Apoe^−/−^ mice [17].

Network pharmacology has emerged as a systems‐level approach to characterise complex drug–target–pathway interactions. By integrating multi‐omics data and biological network analysis, it reveals the polypharmacological actions of natural compounds [18].

Despite progress in AD research, there are still no drugs that directly target its inflammatory and oxidative mechanisms. Given its antioxidant, anti‐inflammatory and anti‐apoptotic activities, morusin may offer vascular protection, yet its mechanism in AD remains unclear.

Therefore, this study focuses on elucidating how morusin exerts protective effects against aortic dissection by regulating inflammation and oxidative stress. We employed an integrated systems biology framework combining network pharmacology, molecular docking, single‐cell transcriptomics and experimental validation to identify its key molecular targets and signalling pathways.

## 2 Methods and Materials

### 2.1 Network Pharmacology Analysis

#### 2.1.1 Identification of AD‐ and Morusin‐Related Target Genes

The SMILES representation of morusin was obtained from the PubChem database (CID: 5281671). To identify potential targets of morusin, multiple public databases were integrated, including TargetNet [19], PharmMapper [20], SuperPred [21] and SwissTargetPrediction [22]. To obtain AD‐related targets, the keyword ‘Aortic dissection’ was used to search the GeneCards database, with a disease target selection criterion of ‘score > 2.0’ to build an AD‐related target dataset. The intersection of the morusin target database and the aortic dissection disease target database was then identified using Venny 2.1.0 to obtain the common targets of both.

#### 2.1.2 Pathway Enrichment Analysis

The identified common targets were imported into the DAVID database for Gene Ontology (GO) enrichment analysis and Kyoto Encyclopedia of Genes and Genomes (KEGG) pathway analysis. To enhance data visualisation, the top 10 terms from the biological process (BP), molecular function (MF) and cellular component (CC) categories in the GO enrichment analysis, as well as the top 20 pathways from the KEGG pathway analysis, were visualised using the Bioinformatics platform [23].

#### 2.1.3 Protein–Protein Interaction (PPI) Network Construction

The PPI network was constructed using the STRING database, and the results were visualised with Cytoscape 3.9.1 [24]. Subsequently, the network topology was analysed using the CytoNCA plugin to identify key components and targets with high degree centrality (DC), betweenness centrality (BC) and closeness centrality (CLC) [25].

#### 2.1.4 Molecular Docking

The molecular docking workflow used in this study follows current computational standards for ligand–protein interaction analysis [26, 27]. The structural information of morusin was obtained from the PubChem database, while the 3D structures of target proteins were retrieved from the Protein Data Bank (PDB). PyMOL 2.4.0 and AutoDockTools 1.5.7 were used for the preprocessing of receptors and ligands, including water molecule removal, hydrogen addition and charge assignment, followed by exporting the structures in *pdbqt* format. Molecular docking was then performed using AutoDock Vina, and the docking results were visualised and analysed using PyMOL.

#### 2.1.5 Collection and Preprocessing of Single‐Cell RNA Sequencing Data

The single‐cell RNA sequencing (scRNA‐seq) dataset for AD was downloaded from the NCBI Gene Expression Omnibus (GEO) under the accession number GSE213740. Data preprocessing was conducted using R Studio, and uniform manifold approximation and projection (UMAP) was applied for visualisation.

Recent research has employed a comprehensive network pharmacology workflow similar to ours. Pasala et al. utilised this strategy to elucidate the cardioprotective mechanism of silybin, identifying key nodes such as AKT1, TNF and IL‐6, as well as pathways including PI3K‐Akt and HIF‐1 [25]. These findings are consistent with the results of our study and confirm the rationality of our approach.

### 2.2 Experimental Vertification

#### 2.2.1 Cell Culture and AngII Intervention

The human aortic vascular smooth muscle cell line (HAVSMC) was obtained from Procell Life Science & Technology Co. Ltd. (Wuhan, China). Cells were maintained in complete growth medium (DMEM supplemented with 10% foetal bovine serum [FBS] and 1% penicillin–streptomycin) at 37°C in a humidified atmosphere containing 5% CO_2_. To establish an in vitro model of vascular inflammation, cells were stimulated with 100 nM AngII [28, 29, 30] (MedChemExpress, Monmouth Junction, NJ, USA).

#### 2.2.2 Morusin Intervention

Morusin (purity ≥ 99%, MedChemExpress, Monmouth Junction, NJ, USA) was dissolved in DMEM to prepare concentration gradients of 0, 1, 3, 6 and 9 μg/mL. Following AngII stimulation (100 nM), HAVSMCs were co‐treated with various concentrations of morusin to evaluate its modulatory effects on vascular smooth muscle cells under inflammatory conditions.

#### 2.2.3 Cell Viability Assessed by CCK‐8 Assay

HAVSMCs were seeded in 96‐well plates at a density of 4 × 10^3^ cells/well and allowed to adhere for 24 h. Cells were then treated with complete medium containing varying concentrations of morusin (0, 1, 3, 6 and 9 μg/mL) for an additional 24 h. Following treatment, 10 μL of CCK‐8 reagent was added to each well, and plates were incubated at 37°C for 1 h. Absorbance was measured at 450 nm using a microplate reader, and cell viability was calculated as a percentage relative to untreated controls.

#### 2.2.4 Quantitative Real‐Time PCR Analysis

First‐strand cDNA synthesis was carried out using a one‐step reverse transcription kit (Cowin Biotech, Beijing, China). Quantitative real‐time PCR (qRT‐PCR) was performed using SYBR Green chemistry on the QuantStudio 3 real‐time PCR system (Thermo, USA). Primer sequences are listed in Table 1.

**TABLE 1:** Primer Sequences for Quantitative Real‐Time PCR (qPCR) (5′‐3′).

Gene	Forward	Reverse
*Hsp90aa1*	CGTTTCTGAGAAGCAGGGCA	GCTGTTTCCAGAGACAGAGTAGAG
*Mapk8*	ACACAACAAACTTAAAGCCAGTCAG	ATCTAACTGCTTGTCAGGGATCTTT
*Mapk1*	GCTGAACCACATTTTGGGTATTCTT	ATGGCACCTTATTTTTGTGTGGAAG
*Ptgs2*	GCAGGCTAATACTGATAGGAGAGAC	ATGCCAGTGATAGAGGGTGTTAAAT
*Mapk14*	GTTACCAGAACCTGTCTCCAGTG	GTCCAACAGACCAATCACATTTTCA
*Nfkb1*	GCAGAAGATGATCCATATTTGGGAA	CGGAAACGAAATCCTCTCTGTTTAG
*Casp3*	GTGGAATTGATGCGTGATGTTTCTA	AGGCCTGAATAATGAAAAGTTTGGG
*Gapdh*	GTCTCCTCTGACTTCAACAGCG	ACCACCCTGTTGCTGTAGCCAA

*Note:* All primer sequences were designed using the NCBI online platform.

### 2.3 Animal Model and Treatment

C57BL/6J male mice (4‐week‐old) were purchased from Charles River Laboratories (Beijing, China). The aortic dissection model was induced by administering 0.25% BAPN in drinking water (freshly prepared weekly) for 4 weeks [31]. Morusin‐treated mice received intragastric administration of morusin (40 mg/kg in saline) twice weekly [17], while vehicles received saline alone. Animals were monitored daily for signs of distress, and humane endpoints were strictly observed: mice showing imminent mortality were immediately euthanised by cervical dislocation, while those surviving to the 4‐week endpoint were euthanised via intravenous pentobarbital sodium overdose (100 mg/kg). Aortic tissues were collected for subsequent analysis.

#### 2.3.1 Ultrasonographic Assessment

Cardiovascular function was evaluated using the Visual Sonics Vevo 3100 system. Mice were anaesthetised via inhalation of 3%–5% isoflurane in an induction chamber, then transferred to a temperature‐controlled platform in the supine position. Electrodes were attached to limbs for continuous electrocardiographic monitoring. High‐frequency linear transducers were employed to acquire longitudinal and transverse aortic views, with optimised gain and depth settings. End‐diastolic aortic internal diameters were measured for functional assessment.

#### 2.3.2 Histology

Aortic tissues were fixed overnight in 4% paraformaldehyde (PFA), embedded in paraffin, and sectioned at 5 μm thickness. Serial sections underwent Verhoeff‐Van Gieson (EVG) and haematoxylin–eosin (HE) staining using standardised protocols, with subsequent microscopic evaluation performed under bright‐field illumination to assess structural integrity and pathological alterations.

### 2.4 Statistical Analysis

**FIGURE 1:**
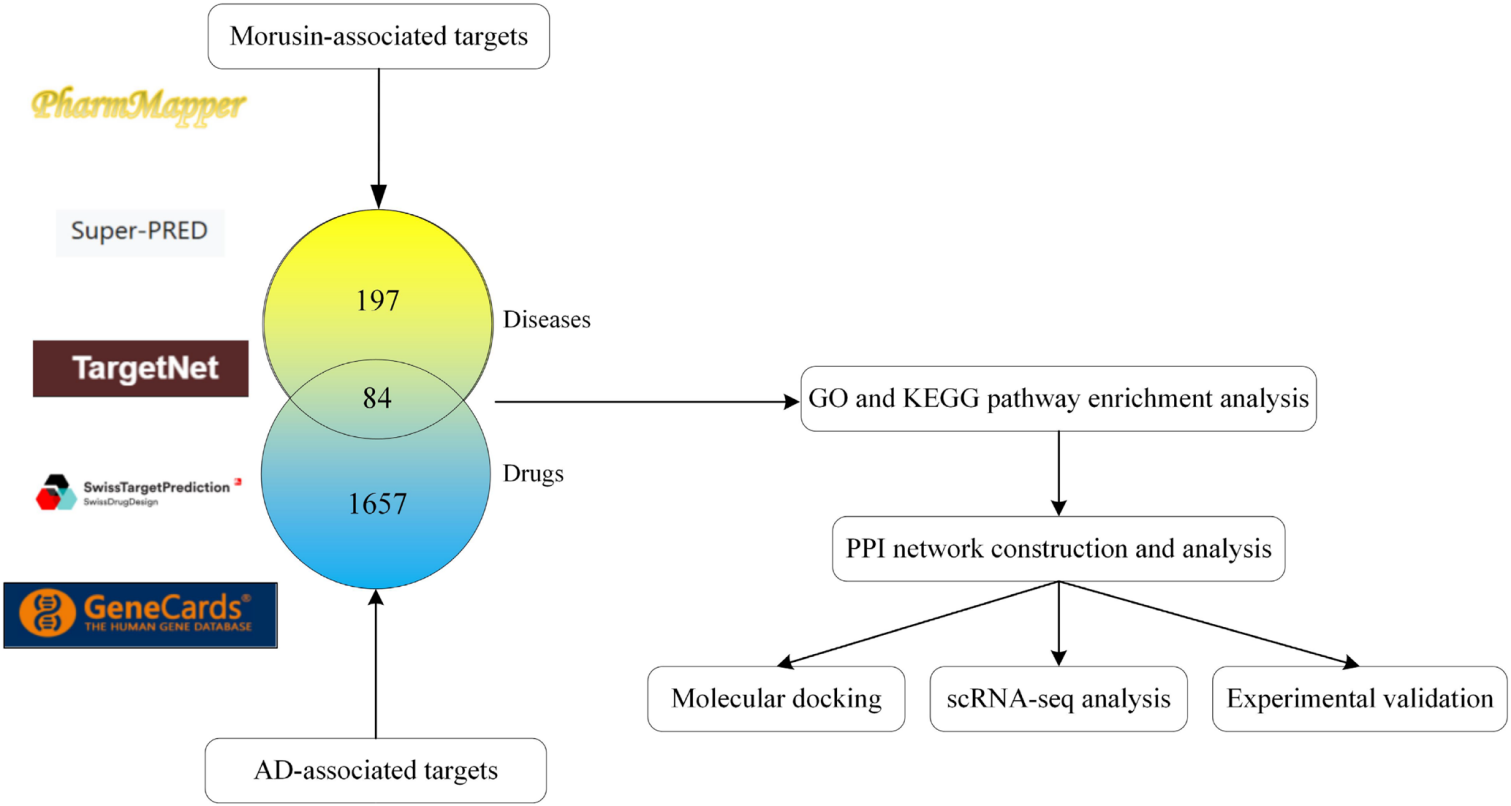
Research workflow diagram of this study.

GraphPad Prism 8.0 was employed for data plotting, while SPSS 25.0 was used for statistical computations. For comparisons between two groups, independent Student's *t*‐tests were conducted to assess statistical significance. When analysing three or more groups, one‐way analysis of variance (ANOVA) was applied based on data characteristics. A threshold of *p* < 0.05 was established for statistical significance. The workflow of the study is summarized in Figure 1.

## 3 Results

### 3.1 Identification of Morusin‐Related Targets

**FIGURE 2:**
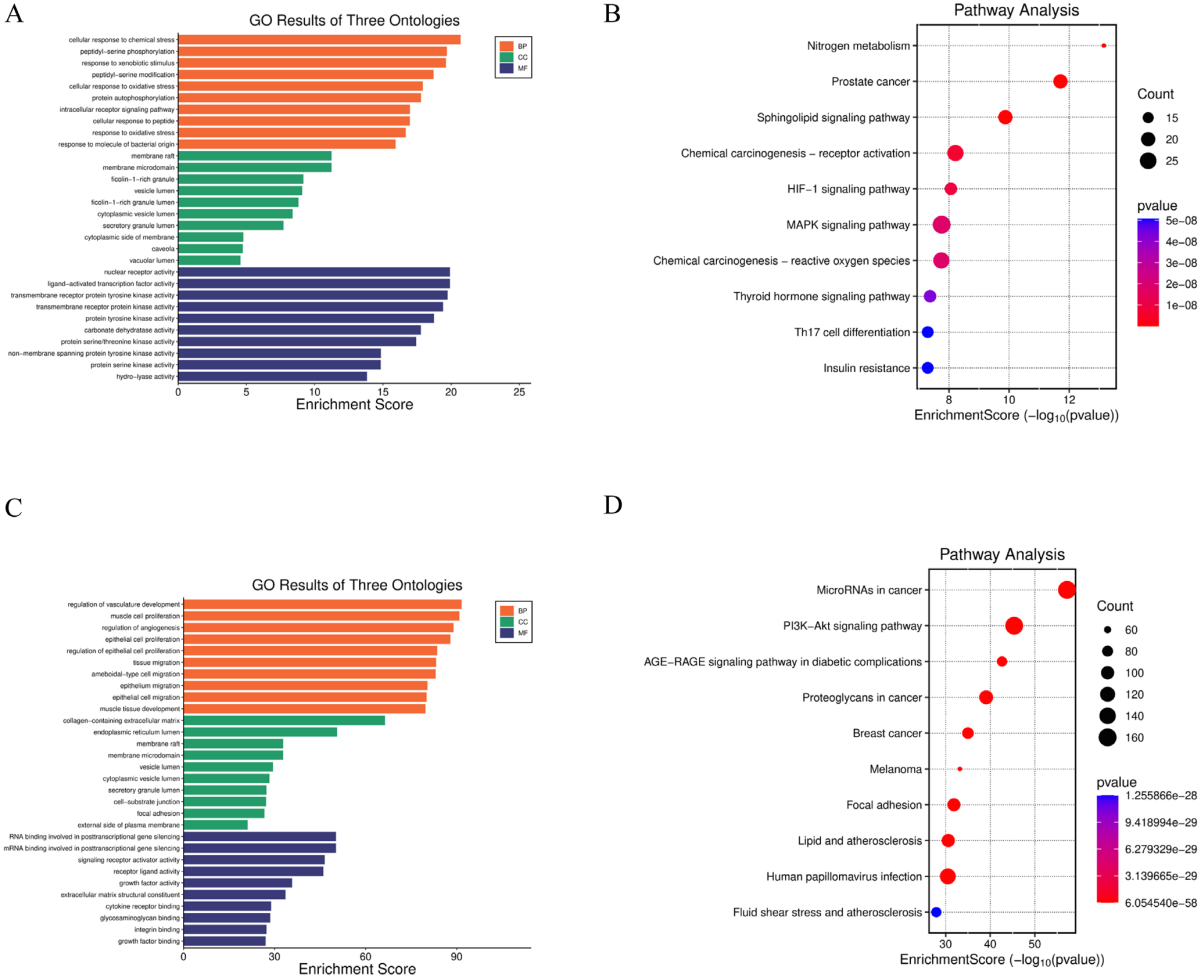
Identification of morusin‐related targets and AD‐related genes. (A) The 10 representative terms with the lowest *p*‐value of biological processes (BPs), cellular components (CCs) and molecular functions (MFs) of the GO enrichment analysis of morusin‐related targets (*p* < 0.05). (B) The 10 significantly enriched KEGG pathways of morusin‐related targets (*p* < 0.05). (C) The 10 representative terms with the lowest *p*‐value of BP, CC and MF of the GO enrichment analysis of AD‐related target genes (*p* < 0.05). (D) The 10 significantly enriched KEGG pathways of AD‐related target genes (*p* < 0.05).

By integrating multiple databases including PharmMapper, SuperPred, TargetNet and SwissTargetPrediction, we systematically identified 281 potential targets of morusin (Table S1). Based on Gene Ontology (GO) functional annotation and KEGG pathway enrichment analysis, we found that the cellular response to chemical stress, membrane raft and nuclear receptor activity were the most significantly enriched terms in the biological process (BP), cellular component (CC) and molecular function (MF) categories, respectively (Figure 2A), suggesting that transcriptional regulation may be involved in its mechanism of action. KEGG pathway enrichment analysis of the top 10 pathways revealed significant enrichment in the sphingolipid signalling pathway, hypoxia‐inducible factor 1 (HIF‐1) signalling pathway, MAPK signalling pathway, among others (Figure 2B).

### 3.2 Identificaiton of AD‐Related Genes

To investigate potential therapeutic targets for aortic dissection (AD), we systematically screened 1741 ad‐related candidate genes using the GeneCards database (Table S2). GO functional annotation and KEGG pathway enrichment analysis revealed that the regulation of vasculature development, collagen‐containing extracellular matrix and RNA binding involved in posttranscriptional gene silencing was the most significantly enriched term in the BP, CC and MF categories, respectively (Figure 2C). Furthermore, KEGG pathway analysis (Top 10 pathways) indicated that the molecular mechanisms underlying AD involve the coordinated interaction of multiple pathways. Notably, the pronounced activation of the PI3K‐Akt signalling pathway may contribute to AD progression by modulating cell survival and vascular remodelling, while the enrichment of pathways related to lipid and atherosclerosis suggests a potential association between metabolic dysregulation and endothelial injury (Figure 2D).

### 3.3 Identification of the Anti‐AD Pathway Analysis of Morusin

**FIGURE 3:**
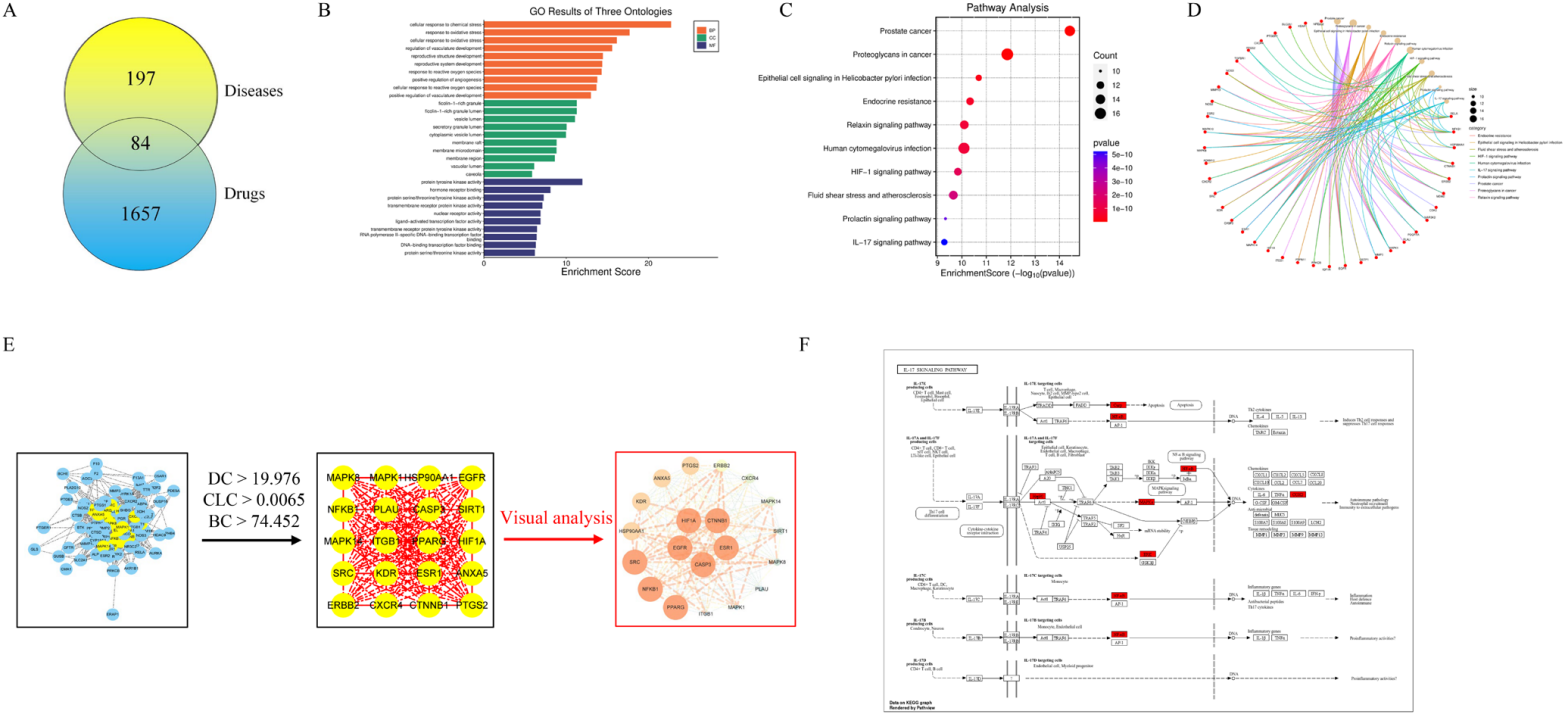
Identification of morusin potential therapeutic pathways. (A) 84 common targets of morusin and AD‐associated targets were visualised by Venn diagram. (B) The 10 representative terms with the lowest *p*‐value of biological processes (BPs), cellular components (CCs) and molecular functions (MFs) of the GO enrichment analysis of the common targets (*p* < 0.05). (C) The 10 significantly enriched KEGG pathways of the common targets (*p* < 0.05). (D) cnet plot of the interactions between the common target genes and KEGG pathways. Red circular nodes represent key molecular targets, while yellow circular nodes denote KEGG pathways. Edges indicate associations between targets and pathways, the size of the nodes represents the quantity of genes. (E) The PPI network of morusin in AD, the screening conditions of hub genes are degree centrality (DC) > 19.976, betweenness centrality (BC) > 74.452 and closeness centrality (CLC) > 0.0065. (F) The position of the core target of morusin in improving AD within the IL‐17 pathway. The red rectangular nodes represent core genes.

By intersecting the identified morusin targets with AD‐related targets, we obtained 84 potential therapeutic targets for morusin in AD using Venny2.1.0 (Figure 3A, Table S3). To further explore the impact of morusin on aortic dissection, we conducted GO enrichment analysis of its potential targets (Figure 3B). The results indicated that, at the BP level, morusin primarily modulates key pathways involved in cellular responses to chemical and oxidative stress, regulation of vasculature development and response to reactive oxygen species, thereby influencing AD progression. In terms of CC, its targets were significantly enriched in compartments such as the lumen of ficolin‐1‐rich granules, vesicle lumens, secretory granule lumens, cytoplasmic vesicle lumens and membrane rafts. Furthermore, MF analysis revealed that morusin may exert its biological effects by modulating protein tyrosine kinase activity, hormone receptor binding, protein serine/threonine/tyrosine kinase activity, transmembrane receptor protein kinase activity and nuclear receptor activity.

KEGG pathway enrichment analysis demonstrated that morusin's target genes were significantly enriched in endocrine resistance, relaxin signalling pathway, HIF‐1 signalling pathway, fluid shear stress and atherosclerosis and the interleukin‐17 (IL‐17) signalling pathway (Figure 3C,D).

### 3.4 Protein–Protein Interaction (PPI) Network Analysis

Based on the interaction network characteristics of morusin and AD, we constructed a PPI network for the core targets using the STRING database and visualised the results with Cytoscape 3.9.1. To further identify key regulatory nodes within the network, we performed topological analysis using the CytoNCA plugin and screened essential targets based on degree centrality (DC), closeness centrality (CLC) and betweenness centrality (BC) metrics. A total of 20 core targets were identified, among which HIF1A, β‐catenin 1 (CTNNB1), epidermal growth factor receptor (EGFR), oestrogen receptor 1 (ESR1) and caspase 3 (CASP3) exhibited significantly higher network centrality (Figure 3E), suggesting that these proteins may serve as key components among the core targets.

### 3.5 Molecular Docking

**FIGURE 4:**
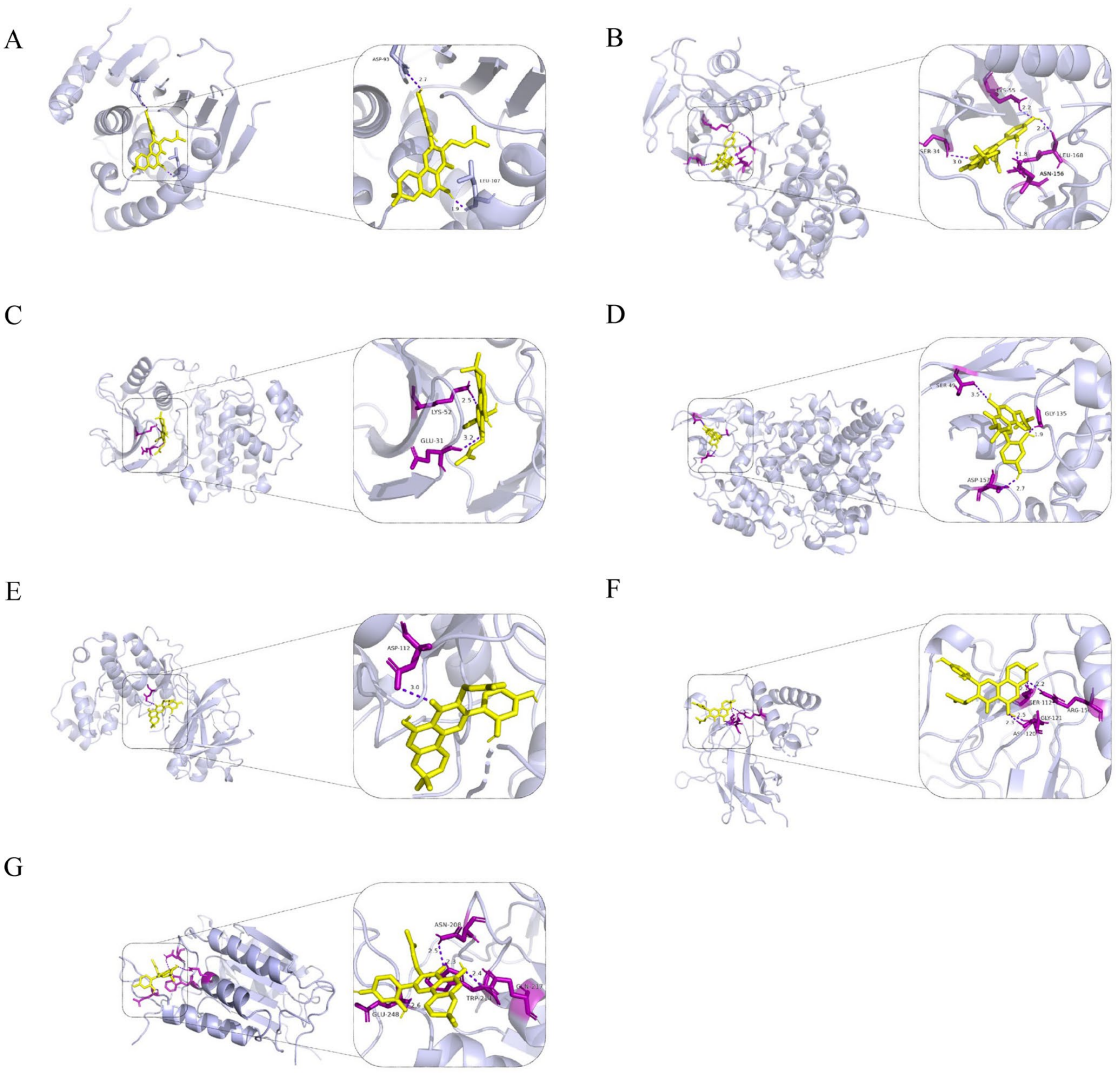
Molecular docking results of morusin and hub targets. (A) Morusin‐HSP90AA1. (B) Morusin‐MAPK8. (C) Morusin‐MAPK2. (D) Morusin‐PTGS2. (E) Morusin‐MAPK14. (F) Morusin‐NFKB1. (G) Morusin‐CASP3.

KEGG pathway enrichment analysis of the 20 core targets revealed significant enrichment in the IL‐17 signalling pathway (Figure S1), with hub targets including *HSP90AA1, MAPK8, MAPK1, MAPK14, PTGS2, NFKB1* and *CASP3* (Figure 3F). To validate the interaction potential between morusin and these hub targets, we performed molecular docking simulations using AutoDock Vina (Figure 4). Consistent with these recent computational frameworks, our docking analysis identified stable ligand–protein binding conformations and quantified binding affinities. The results demonstrated high‐affinity binding between morusin and all seven targets, with binding energies ranging from −9.0 to −7.0 kcal/mol (Table 2).

**TABLE 2:** Interaction parameters of 20 hub targets and morusin.

Protein	PDB ID	affnity (kcal/mol)	RMSD_Ib (Å)	RMSD_ub (Å)
HSP90AA1	1uyd	−9.0	2.103	6.285
MAPK8	4qtd	−8.8	2.083	4.346
EGFR	5ug8	−8.5	4.595	11.218
ERBB2	1 s78	−8.4	1.879	8.347
MAPK1	2oji	−8.4	2.264	4.795
PTGS2	5kir	−8.3	1.619	2.189
CXCR4	8k3z	−8.1	2.326	8.445
SIRT1	4zzh	−8.1	2.772	3.767
PLAU	1owe	−8.0	4.917	9.544
PPARG	1zgy	−7.9	2.196	5.214
ANXA5	8h9z	−7.7	2.468	6.909
CTNNB1	1p22	−7.7	6.910	8.889
MAPK14	2qd9	−7.5	2.360	6.925
KDR	1vr2	−7.4	1.501	2.101
ITGB1	3t9k	−7.3	22.769	25.249
NFKB1	8tqd	−7.1	14.872	19.115
CASP3	5ibp	−7.0	6.940	12.282
ESR1	2iog	−7.0	15.203	17.662
HIF1A	4h6j	−6.4	5.834	10.188
SRC	1a1c	−6.2	23.796	26.257

*Note:* PDB (Protein Data Bank) is a structural database of proteins; RMSD (Root‐Mean‐Square Deviation) quantifies the spatial positional differences of heavy atoms (non‐hydrogen atoms) between conformations relative to the optimal conformation.

### 3.6 Single‐Cell RNA‐Seq Reveals Morusin's Protection Against Aortic Dissection

**FIGURE 5:**
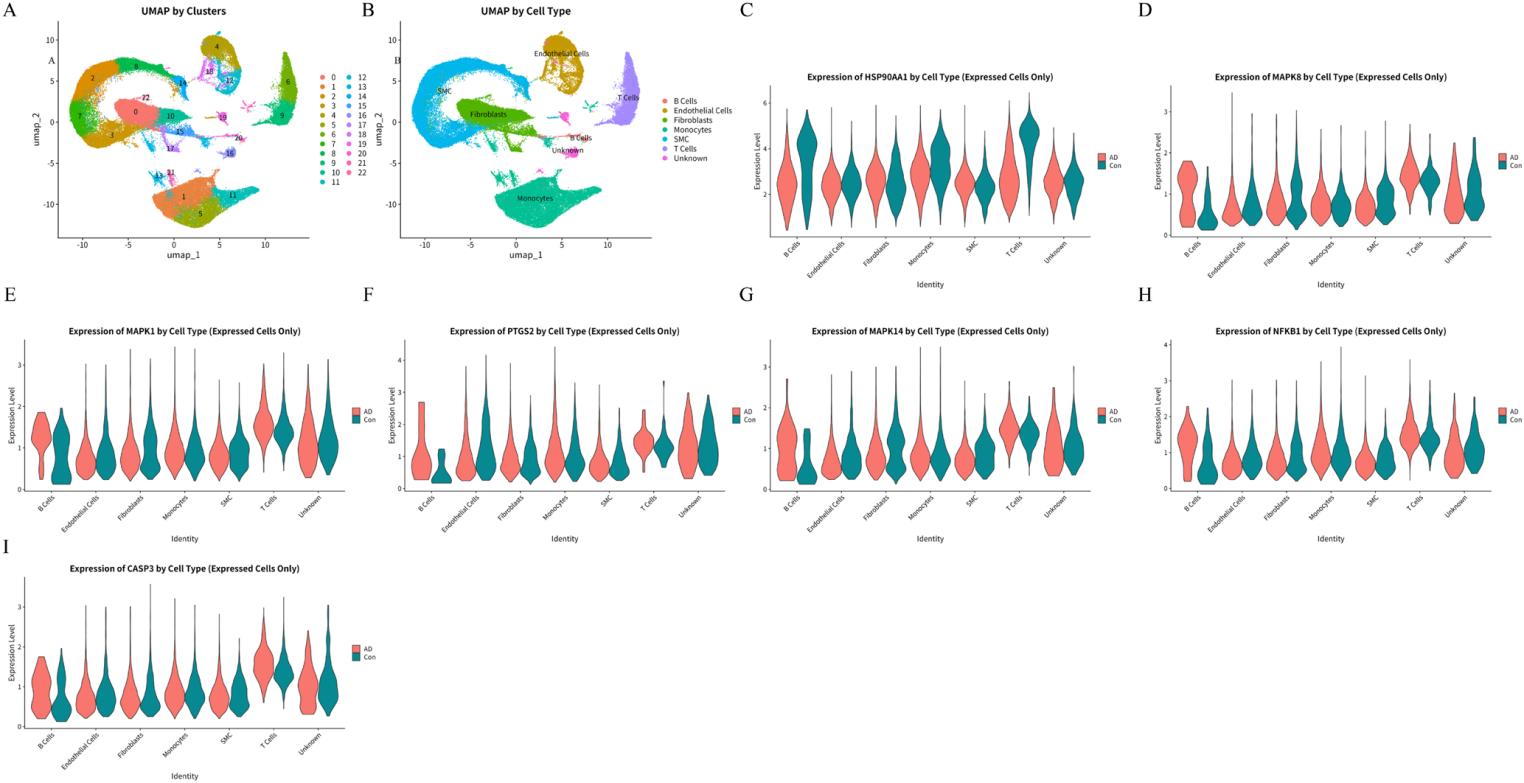
The hub targets expression in different AD cell types. (A, B) UMAP plots show 22 clusters and 6 cell types in AD patients. (C–I) The hub targets expression in different cell types.

To further investigate the expression differences of core target genes across various cell types, we analysed single‐cell transcriptomic data from human aortic dissection tissues (GSE213740) obtained from the GEO database. A total of 22 distinct cell clusters were identified and classified into 6 major cell types based on marker gene expression: B cells, endothelial cells, fibroblasts, monocytes, smooth muscle cells and T cells (Figure 5A,B).

Gene expression analysis across these cell types revealed significant differential expression patterns of hub target genes (Figure S2). Notably, HSP90AA1 was significantly downregulated in T cells and B cells from AD tissues (Figure 5C). In contrast, MAPK8, MAPK1, PTGS2 and MAPK14 were markedly upregulated in fibroblasts, B cells and T cells (Figure 5D–G), while their expression remained relatively unchanged in smooth muscle and endothelial cells. Furthermore, NFKB1 exhibited selective expression in T cells (Figure 5H), whereas CASP3 showed relatively low expression in fibroblasts (Figure 5I).

### 3.7 In Vitro and In Vivo Validation of Morusin's Protection Against Aortic Dissection

**FIGURE 6:**
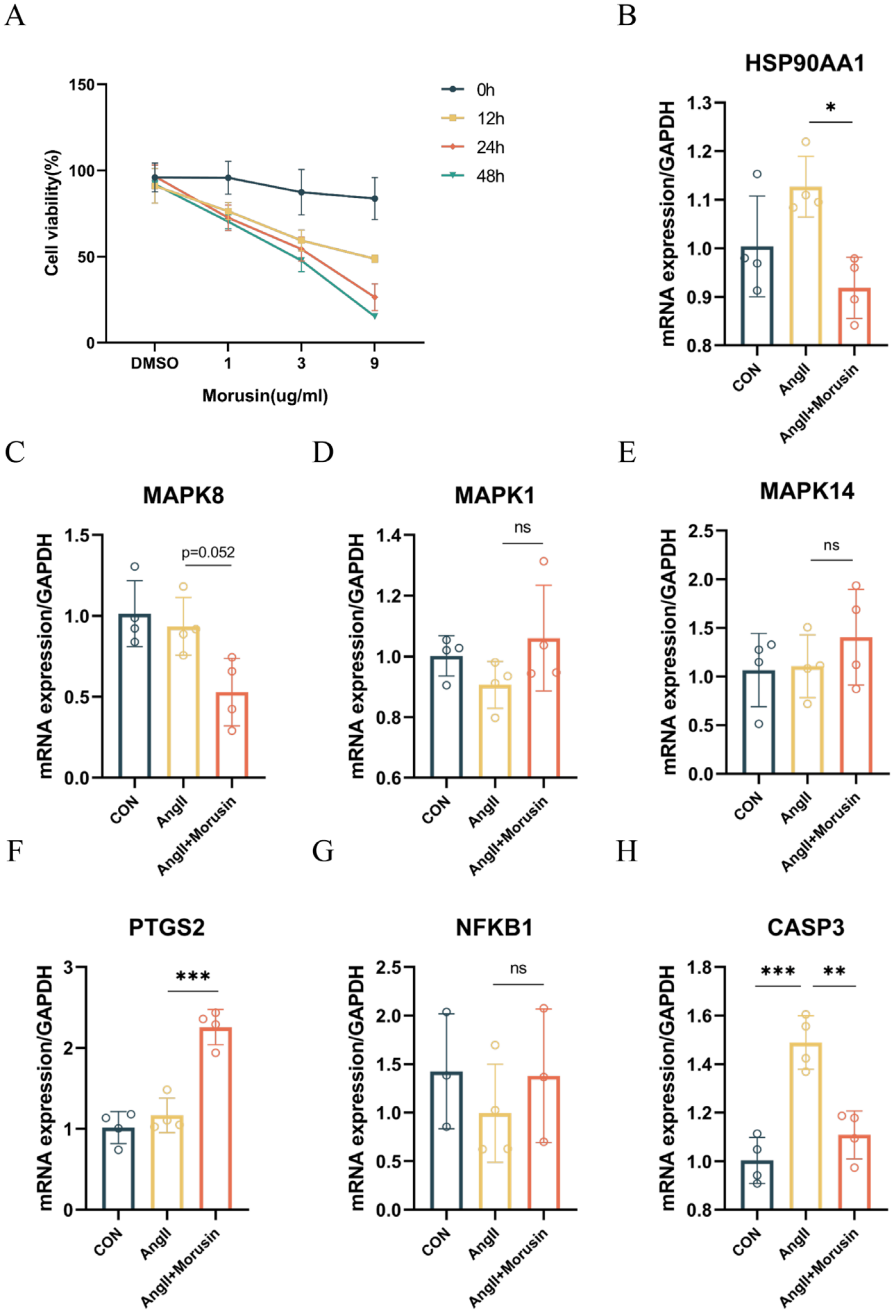
Effects of Morusin Intervention on IL‐17 Pathway‐Related mRNA Levels and Cell Viability in HAVSMCs. (A) Effect of morusin on the viability of HAVSMCs. (B) Changes in HSP90AA1 gene expression levels. *p < 0.05. (C) Changes in MAPK8 gene expression levels. (D) Changes in MAPK1 gene expression levels. (E) Changes in MAPK14 gene expression levels. (F) Changes in PTGS2 gene expression levels. ***p < 0.001. (G) Changes in NFKB1 gene expression levels. (H) Changes in CASP3 gene expression levels. **p < 0.01, ***p < 0.001 (*n* = 4).

Next, we established an in vitro aortic dissection model by stimulating HAVSMC with 100 nM AngII. Morusin treatment (0–9 μg/mL) dose‐ and time‐dependently inhibited HAVSMC proliferation, achieving maximal suppression > 80% at 9 μg/mL after 48 h (Figure 6A). Based on our prior bioinformatics analysis that highlighted the potential regulatory role of the IL‐17 signalling pathway, we measured the mRNA expression levels of key related genes. The data revealed that morusin treatment (3 μg/mL for 24 h) significantly downregulated the pro‐inflammatory mediators HSP90AA1, MAPK8 and CASP3 (Figure 6B,C,H), while upregulating the anti‐inflammatory marker PTGS2 (Figure 6F).

**FIGURE 7:**
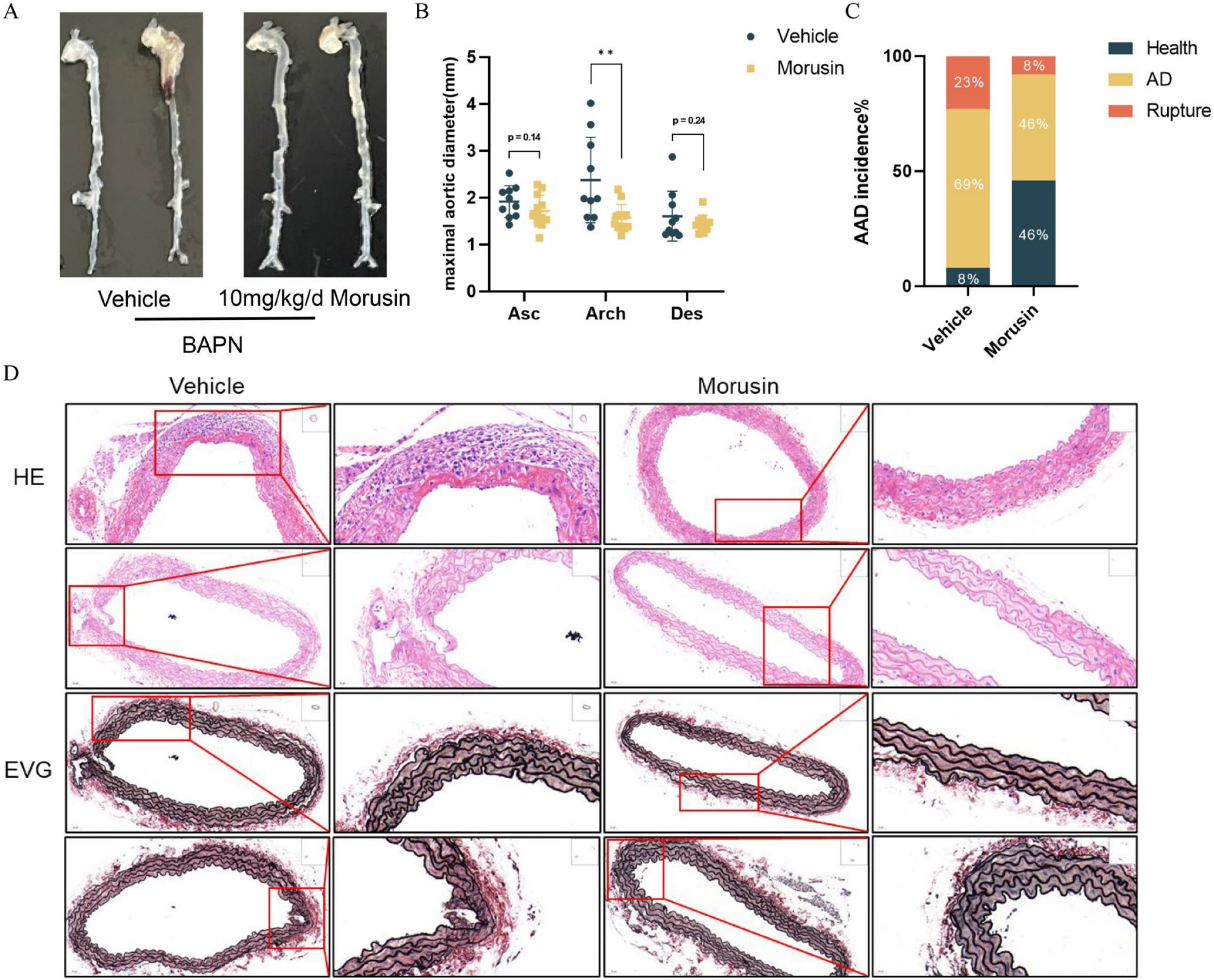
Supplementation with Morusin attenuates aortic dissection in vivo. (A) Four‐week‐old male mice were administered with 0.25% BAPN (wt/vol) for 30days with or without morusin (40 mg/kg in saline): Vehicle + BAPN, *n* = 13; and morusin + BAPN, *n* = 13. (B) Maximal aortic diameter measurement of each group. **p < 10 vehicle + BAPN, *n* = 10; morusin + BAPN, *n* = 12. (C) Incidence of aortic dissection for each group. (D) Representative images of haematoxylin and eosin (20X: Scale bar = 50 μm; 40X: Scale bar = 20 μm) and Elastic Van Gieson (20X: Scale bar = 50 μm; 40X: Scale bar = 20 μm) staining in the thoracic aorta of mice.

To evaluate the therapeutic efficacy of morusin, we established an aortic dissection model in mice using BAPN administration. At day 28, ultrasonographic examination of the aortic root revealed significantly attenuated aortic dilation in the morusin‐treated group compared to vehicle controls (Figure 3). Subsequent terminal harvest at day 30 demonstrated marked mitigation of aortic arch expansion in morusin‐treated mice, with concomitant reductions in dilation severity throughout ascending and descending aortic segments (Figure 7A,B). Quantitative analysis further indicated that morusin intervention reduced both dissection incidence (46% vs. 69% in the vehicle group) and mortality (8% vs. 23%) (Figure 7C). Histopathology confirmed morusin's protective effects: vehicle‐treated aortas displayed hematoma, neointimal hyperplasia, elastic fibre fragmentation and structural disintegration, whereas morusin preserved vascular integrity with minimal pathology (Figure 7D).

## 4 Discussion

Aortic dissection is a fatal cardiovascular emergency caused by a tear in the aortic intima or the formation of an intramural hematoma, leading to the separation of the aortic media. Although the application of thoracic endovascular aortic repair (TEVAR) and antihypertensive medications has partially improved patient outcomes [32], specific therapeutic agents targeting core pathological mechanisms—such as vascular wall matrix degradation and inflammatory cascade reactions—are still lacking in clinical practice. Consequently, natural products with multi‐target regulatory properties have emerged as a promising new direction in AD treatment research.

Morusin, an allylated flavonoid isolated from the root bark of Morus species, has demonstrated anti‐inflammatory, antioxidant and anti‐apoptotic properties in various disease models, including diabetes [9], cancer [33] and cardiovascular calcification [17]. In view of these findings, the present study aims to elucidate the potential molecular targets and pathways through which morusin modulates AD by integrating network pharmacology, molecular docking and in vitro experimental validation.

This study focused on uncovering the multi‐target and multi‐pathway mechanisms by which morusin mitigates AD progression, linking computational predictions to biological validation. Through a multi‐database integration strategy, we identified 281 potential targets of morusin and 1741 ad‐related targets. GO enrichment and KEGG pathway analyses revealed that the IL‐17 signalling pathway, HIF‐1 signalling pathway and MAPK signalling pathway are the three core pathways through which morusin modulates AD. Further topological analysis of the PPI network identified seven key hub genes among the 84 overlapping targets: HSP90AA1, MAPK8, MAPK1, MAPK14, PTGS2, NFKB1 and CASP3, all of which exhibited stable ligand‐receptor binding conformations in molecular docking simulations.

Single‐cell transcriptomic analysis further revealed cell‐type‐specific expression patterns of these targets within the AD tissue microenvironment: HSP90AA1 was significantly downregulated in T/B lymphocytes, whereas MAPK8/1/14 were co‐activated in fibroblasts and immune cells. These findings suggest that morusin may inhibit vascular wall inflammatory damage by modulating immune‐stromal cell interaction networks. Our single‐cell data also provided further characterisation of endothelial cell heterogeneity and functional states in AD tissues. Endothelial dysfunction represents a pivotal contributor to AD pathogenesis, and AngII, the stimulant employed in our in vitro model, is a well‐established inducer of such dysfunction [34]. A central mechanism through which AngII compromises endothelial function involves the activation of a detrimental signalling cascade. This cascade is marked by the upregulation of von Willebrand Factor (vWF), which subsequently enhances the activation of NADPH oxidase isoforms (NOX2/4), thereby elevating oxidative stress levels and ultimately triggering the overexpression of Endothelin‐1 (ET‐1) [35, 36]. This pathological axis of disease leads to marked vasoconstriction, sustained inflammatory responses and extracellular matrix degradation—hallmarks of AD progression [37, 38]. Although the present validation primarily focused on VSMCs, network pharmacology analysis revealed that the HIF‐1 signalling pathway is particularly responsive to oxidative stress and plays a crucial role in maintaining endothelial homeostasis, thereby representing a promising therapeutic target for morusin. The potential antioxidant and anti‐inflammatory effects of morusin may confer protective benefits on the endothelium by modulating the vWF/NOX/ET‐1 axis. Future studies should aim to directly evaluate the capacity of morusin to regulate endothelial vWF expression and ET‐1 secretion, which could offer novel insights into its therapeutic mechanisms.

In vitro, using the HAVSMC cell line, we demonstrated that morusin significantly reduced cell viability in a dose‐dependent manner following Ang II stimulation. In vivo, morusin effectively attenuated thoracic aortic dilation and decreased the morbidity and mortality associated with aortic dissection in a BAPN‐induced mouse model. Simultaneously, analysis of mRNA expression related to the IL‐17 pathway revealed that morusin treatment led to a decrease in the expression of HSP90AA1, MAPK8 and CASP3, while the expression of PTGS2 increased. HSP90AA1, a molecular chaperone, stabilises the conformation of vascular stress response proteins, thereby alleviating hypoxia‐induced vascular remodelling disorders. Previous studies have demonstrated that HSP90AA1 promotes autophagy via the PI3K/Akt/mTOR pathway and inhibits apoptosis through the JNK/P38 pathway [39]. Furthermore, single‐cell analyses showed that HSP90AA1 is significantly downregulated in T/B lymphocytes within AD tissues, suggesting that morusin may suppress the activation of inflammatory pathways such as NF‐κB by mitigating the stress response of immune cells. MAPK8, a key kinase in the JNK signalling cascade, phosphorylates the c‐Jun/AP‐1 transcription complex to drive the abnormal proliferation of VSMCs and the expression of pro‐inflammatory mediators, such as IL‐6 and MCP‐1 [40, 41]. Our study found that AngII stimulation markedly upregulated MAPK8 expression in HAVSMCs, an effect that was effectively attenuated by morusin treatment. CASP3 exhibits a dual role in AD: it cleaves Gasdermin E to trigger pyroptosis in VSMCs [42] and the subsequent release of pro‐inflammatory cytokines, including IL‐1β [43], while also participating in the apoptotic clearance of damaged cells [44]. Notably, morusin treatment reduced CASP3 expression by approximately 2.5‐fold, indicating that it may alleviate inflammatory injury in the vascular wall by modulating the balance between pyroptosis and apoptosis.

Although this study systematically elucidates the multi‐target mechanism of morusin, certain limitations remain. Future research should further validate these target regulatory effects in an *Apoe* ^*−/−*^ mouse model of AD and employ conditional gene knockout models to dissect the specific roles of these targets in distinct cell types.

## 5 Conclusion

In conclusion, our study reveals that morusin exerts protective effects against aortic dissection through the modulation of multiple molecular targets and signalling pathways, particularly IL‐17, HIF‐1 and MAPK. By integrating network pharmacology, molecular docking, single‐cell transcriptomics and in vitro/in vivo validation, we demonstrate that morusin can attenuate vascular inflammation, inhibit abnormal VSMC proliferation and regulate cell death processes. These findings provide mechanistic insights into the multi‐target therapeutic potential of morusin and support its development as a promising candidate for future anti‐AD interventions.

## Author Contributions

**Lemin Zheng:** conceptualization (lead), funding acquisition (equal), supervision (equal), writing – review and editing (lead). **Zhaomeng Wang:** validation (equal), writing – original draft (equal). **Haoran Zhang:** data curation (equal), methodology (equal). **Zhanxiong Xie:** methodology (equal), visualization (equal). **Yukun Xiang:** formal analysis (equal), methodology (equal). **Yiwen Fu:** investigation (equal). **Zixun Wang:** methodology (equal). **Haiqing Jiao:** data curation (equal). **Nan Lin:** methodology (equal). **Chenguang Niu:** conceptualization (equal), funding acquisition (equal), writing – review and editing (equal). **Chao Jiang:** conceptualization (equal), funding acquisition (equal), writing – review and editing (equal).

## Funding

This work was supported by the National High Technology Research and Development Program of China (Grant No. 2020YFA0803700; No. 2023YFA1800904); the Natural Science Foundation of Beijing, China (Grant No. 232031 L232031; No. J230039); the Beijing Physician Scientist Training Project (Grant BJPSTP‐2024‐21); the Henan Provincial Science and Technology Research and Development Project (Grant No. 252102311099); the Kaifeng Municipal Science and Technology Research and Development Project (Grant No. 2203023).

## Conflicts of Interest

The authors declare no conflicts of interest.

## Supporting information


**Figure S1:** The 10 significantly enriched KEGG pathways of the 20 core targets (*p* < 0.05).

**Figure S2:** The UMAP plots of hub target expression in different AD and control groups. (A) HSP90AA1 (B) MAPK8 (C) MAPK1 (D) MAPK14 (E) PTGS2 (F) NFKB1 (G) CASP3.

**Figure S3:** Ultrasonographic Examination of the Aortic Root in C57BL/6J Mice.

**Table S1:** jcmm70971‐sup‐0004‐TableS1.xlsx.

**Table S2:** jcmm70971‐sup‐0005‐TableS2.xlsx.

## Data Availability

The data that support the findings of this study are available from the corresponding author upon reasonable request.
