# Supplementary figures and images for: Integrated Network Pharmacology, Single‐Cell Transcriptomics Unveil the Mechanistic Role of Morusin in Aortic Dissection
Source: J Cell Mol Med. 2026 Jan 2;30(1):e70971. doi: 10.1111/jcmm.70971 (PMC12759265; doi:10.1111/jcmm.70971)

## Slide 1
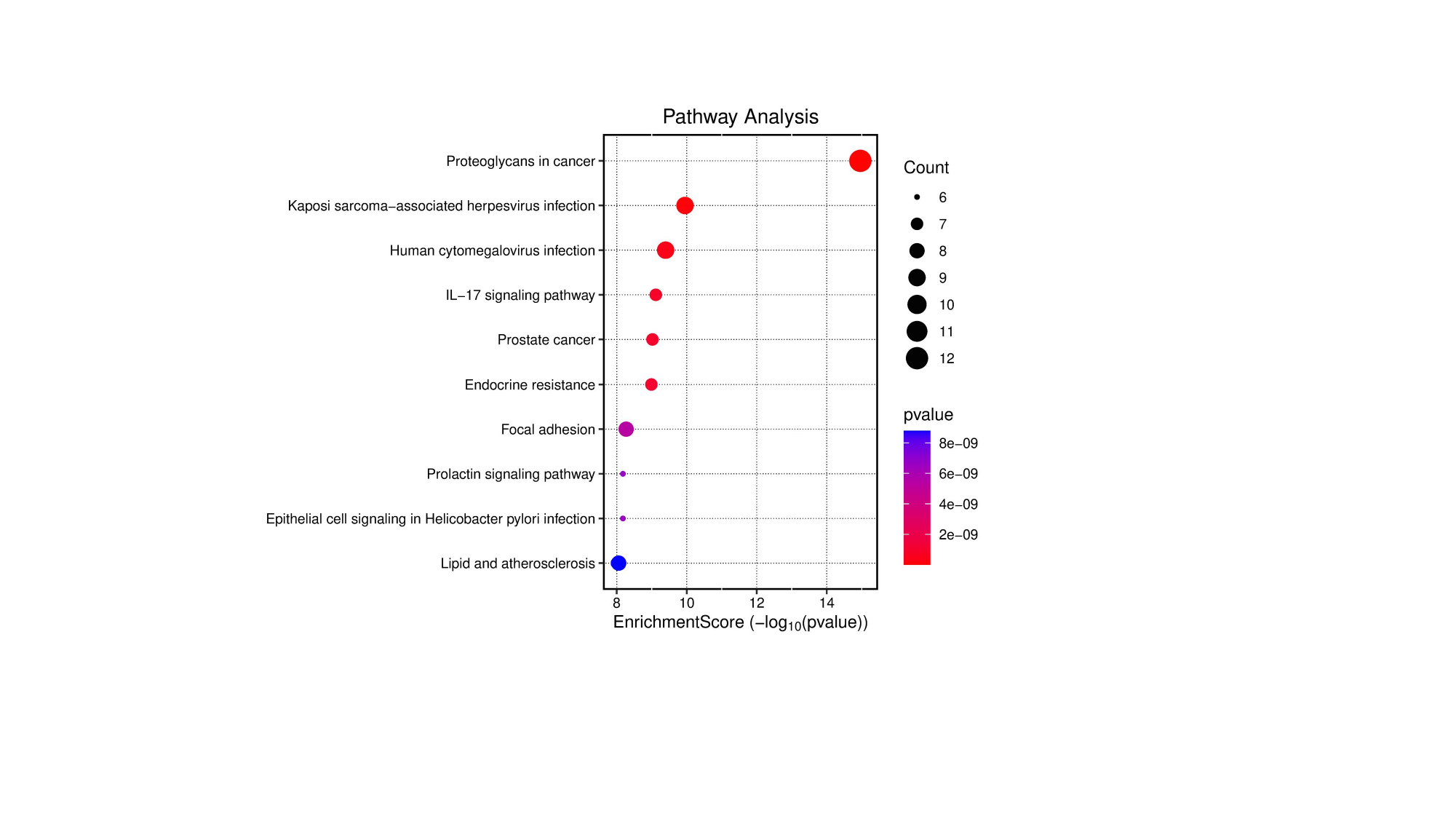

Supplement: Supplementary file 1 — Figure S1: The 10 significantly enriched KEGG pathways of the 20 core targets (p < 0.05). [file JCMM-30-e70971-s003.pptx]

## Slide 1
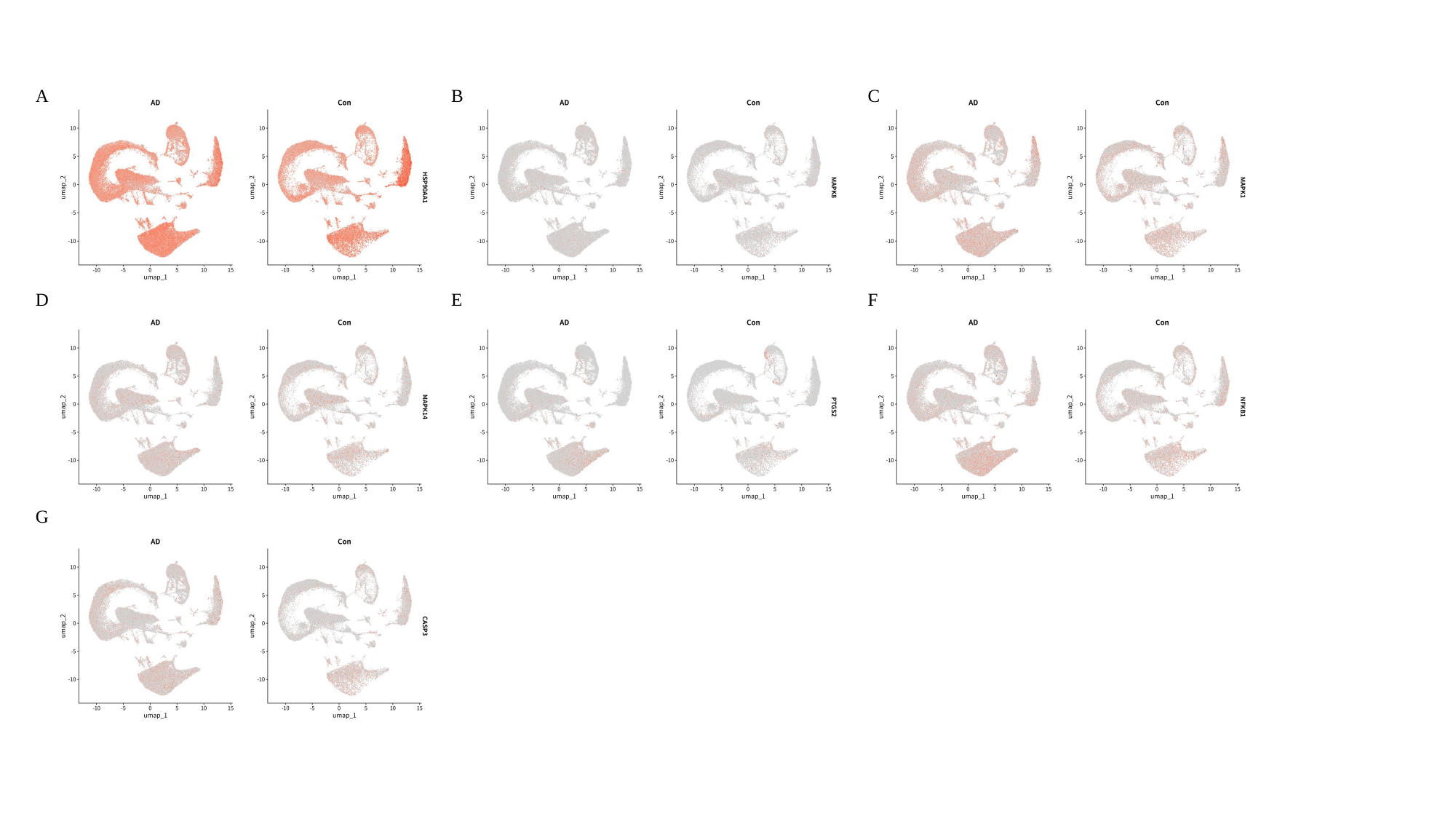

A
B
C
D
E
F
G

Supplement: Supplementary file 2 — Figure S2: The UMAP plots of hub target expression in different AD and control groups. (A) HSP90AA1 (B) MAPK8 (C) MAPK1 (D) MAPK14 (E) PTGS2 (F) NFKB1 (G) CASP3. [file JCMM-30-e70971-s005.pptx]

## Slide 1
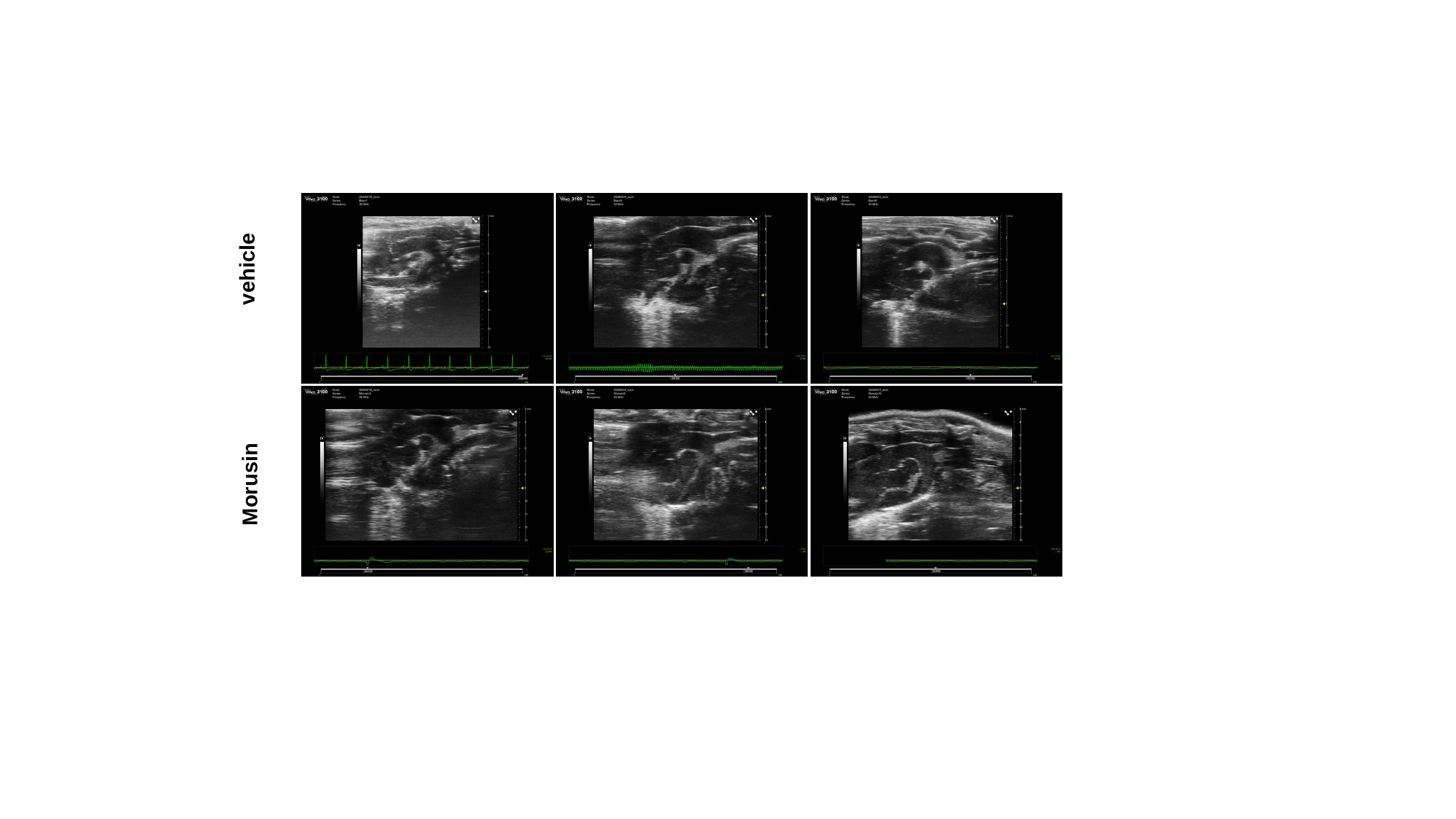

vehicle
Morusin

Supplement: Supplementary file 3 — Figure S3: Ultrasonographic Examination of the Aortic Root in C57BL/6J Mice. [file JCMM-30-e70971-s002.pptx]
